# Integrated bioinformatics-based identification of diagnostic markers in Alzheimer disease

**DOI:** 10.3389/fnagi.2022.988143

**Published:** 2022-11-10

**Authors:** Danmei Chen, Yunpeng Zhang, Rui Qiao, Xiangyu Kong, Hequan Zhong, Xiaokun Wang, Jie Zhu, Bing Li

**Affiliations:** ^1^Research Center for Clinical Medicine, Jinshan Hospital Affiliated to Fudan University, Shanghai, China; ^2^Department of Integrative Medicine, Huashan Hospital Affiliated to Fudan University, Shanghai, China; ^3^Department of Neurology, Jinshan Hospital, Fudan University, Shanghai, China; ^4^College of Acupuncture-Massage and Rehabilitation, Yunnan University of Traditional Chinese Medicine, Kunming, China; ^5^Department of Rehabilitation, Jinshan Hospital, Fudan University, Shanghai, China

**Keywords:** Alzheimer’s disease, diagnosis biomarkers, immune infiltration, machine learning, bioinformatics analysis

## Abstract

Alzheimer disease (AD) is a progressive neurodegenerative disease resulting from the accumulation of extracellular amyloid beta (Aβ) and intracellular neurofibrillary tangles. There are currently no objective diagnostic measures for AD. The aim of this study was to identify potential diagnostic markers for AD and evaluate the role of immune cell infiltration in disease pathogenesis. AD expression profiling data for human hippocampus tissue (GSE48350 and GSE5281) were downloaded from the Gene Expression Omnibus database. Differentially expressed genes (DEGs) were identified using R software and the Human Protein Atlas database was used to screen AD-related DEGs. We performed functional enrichment analysis and established a protein–protein interaction (PPI) network to identify disease-related hub DEGs. The fraction of infiltrating immune cells in samples was determined with the Microenvironment Cell Populations-counter method. The random forest algorithm was used to develop a prediction model and receiver operating characteristic (ROC) curve analysis was performed to validate the diagnostic utility of the candidate AD markers. The correlation between expression of the diagnostic markers and immune cell infiltration was also analyzed. A total of 107 AD-related DEGs were screened in this study, including 28 that were upregulated and 79 that were downregulated. The DEGs were enriched in the Gene Ontology terms GABAergic synapse, Morphine addiction, Nicotine addiction, Phagosome, and Synaptic vesicle cycle. We identified 10 disease-related hub genes and 20 candidate diagnostic genes. Synaptophysin (*SYP*) and regulator of G protein signaling 4 (*RGS4*) (area under the ROC curve = 0.909) were verified as potential diagnostic markers for AD in the GSE28146 validation dataset. Natural killer cells, B lineage cells, monocytic lineage cells, endothelial cells, and fibroblasts were found to be involved in AD; additionally, the expression levels of both *SYP* and *RGS4* were negatively correlated with the infiltration of these immune cell types. These results suggest that *SYP* and *RGS4* are potential diagnostic markers for AD and that immune cell infiltration plays an important role in AD development and progression.

## Introduction

Alzheimer disease (AD) is the leading cause of dementia, which is among the most costly, fatal, and burdensome diseases affecting the global population. The estimated worldwide prevalence of dementia in 2018 was 50 million people, which is expected to triple by 2050 (Hodson, 2018). AD is clinically diagnosed and characterized by amyloid beta (Aβ) and tau deposition and loss of synapses in many brain regions including the hippocampus (Jack and Holtzman, 2013). However, there are few objective measurements for the clinical diagnosis of AD; moreover, the diagnosis is often inaccurate and occurs when the disease is already at an advanced stage. Although it is possible to detect Aβ plaques and tau-related neurodegeneration by imaging and from analytes in the cerebrospinal fluid (Jack and Holtzman, 2013), about 40% of nondemented normal elderly people show AD neuropathology including senile plaques and neurofibrillary tangles (Jack and Holtzman, 2013); conversely, some clinically diagnosed AD cases exhibit no neuropathologic features of the disease (Beach et al., 2012). As such, there is a need for precise tools for the early detection of AD.

Immune cell infiltration has been shown to be involved in the onset and progression of AD; dysregulation of innate and adaptive immunity lead to a systemic failure of immune cell-mediated Aβ clearance (Li et al., 2021). Apolipoprotein E (*APOE*), inositol polyphosphate-5-phosphatase D (*INPP5D*), cluster of differentiation 33 (*CD33*), and phospholipase C gamma 2 (*PLCG2*) have been identified as high-risk genes in AD and have been linked to Aβ accumulation in AD mouse models (Sierksma et al., 2020). Natural killer (NK) cell infiltration in the peripheral blood has been observed in AD patients, which may contribute to neuroinflammation (Lu et al., 2021). The microglia receptors CD33 and triggering receptor expressed on myeloid cells 2 (TREM2) regulate neuroinflammation and are therapeutic targets in AD (Naj et al., 2011; Guerreiro et al., 2013; Jonsson et al., 2013). Given the association between immune infiltration and AD pathogenesis, there is potential application for immunotherapy in the treatment of AD. However, the types of infiltrating immune cells and their precise role in AD development are unclear. Identifying the different subsets of immune cells can provide insight into the molecular basis of AD pathology, which can in turn lead to the development of novel treatments.

The aim of this study was to identify novel biomarkers for the early diagnosis of AD and evaluate their contribution to the immune microenvironment of AD using an *in silico* approach. Advancements in computer science have accelerated bioinformatics. As an essential part of artificial intelligence, machine learning has been integrated into many fields, as well as in medicine. Here, we trained randomForest model for predicting the onset of AD, and further identified diagnostic markers for AD. To explore the specific role of these potential markers in the occurrence and development of AD, we conducted immune infiltration analysis and correlation analysis to explain the pathogenesis of AD from the perspective of immune microenvironment.

## Materials and methods

### Data source and preprocessing

The AD datasets GSE48350 (Berchtold et al., 2008), GSE5281 (Liang et al., 2007), and GSE28146 (Liang et al., 2007) from GPL570 were downloaded from the NCBI Gene Expression Omnibus database^1^ (Barrett et al., 2013). Hippocampus samples of 29 AD patients and 56 normal controls were extracted from GSE48350 and GSE5281 dataset. Hippocampus samples from GSE28146 was used for validation. Raw data were read using the “affy” package (Gautier et al., 2004) of R v3.6.1 software^2^ and the rate monotonic algorithm was used for background correction and data normalization. The GSE48350 and GSE5281 gene expression matrices were then combined, and the “sva” package (Parker et al., 2014) was used to detect and eliminate batch effects from the two datasets. A box plot was generated to visualize the effect of removing inter-batch differences, and the effect of inter-sample correction was assessed using a two-dimensional principal component analysis (PCA) cluster plot. Differentially expressed genes (DEGs) were screened with the “limma” package (Ritchie et al., 2015) and a volcano map of DEGs was constructed using the “ggplot2” package to visualize the genes. DEGs with an adjusted *p* value (false discovery rate [FDR]) <0.05 and |log2 fold change (FC)| >0.585 were considered significant. We downloaded the disease-related gene list from the Human Protein Atlas (HPA) protein annotation database (Uhlén et al., 2015) and evaluated the intersection between disease-related genes and DEGs to identify those specifically related to AD.

### Functional enrichment analysis

The “clusterProfiler” package of R software (Yu et al., 2012) was used to perform Gene Ontology (GO) and Kyoto Encyclopedia of Genes and Genomes (KEGG) enrichment analyses of the AD-related DEGs. We used the same package to perform gene set enrichment analysis (GSEA) of the gene expression matrix with “c2.cp.kegg.v7.0.symbols.gmt” as the reference gene set. An FDR <0.25 and *p* value <0.05 were considered as reflecting significant enrichment.

### Construction of protein–protein interaction network and identification of hub genes

The Search Tool for the Retrieval of Interacting Genes (STRING) database^3^ was used to establish PPI network of AD-related DEGs (Szklarczyk et al., 2019). Cytoscape 3.7.1 was utilized to visualize connections with combined score > 0.5. And molecular Complex Detection (MCODE) plugin in Cytoscape was used to identified functional modules in the PPI network with a node score cutoff = 0.2, degree cutoff = 2, k score = 2, and max depth = 100 (Bader and Hogue, 2003). We performed cytoHubba on Cytoscape to screen the top10 node genes by the MCC, EPC and Degree methods (Chin et al., 2014).

### Immune cell infiltration analysis

The R “MCP-counter” package (Becht et al., 2016) was used to analyze the relative proportions of 8 types of infiltrating immune cell (total T cells, CD8^+^ T cells, cytotoxic lymphocytes, NK cells, B lineage cells, monocytic lineage cells, myeloid dendritic cells, and neutrophils) and 2 types of stromal cell (endothelial cells and fibroblasts) in each group. The Microenvironment Cell Populations (MCP)-counter algorithm, which estimates the number of infiltrated immune and nonimmune stromal cells in individual samples by quantifying cell-specific transcript levels, and the R “pheatmap” package^4^ were used to visualize the abundance of tissue-infiltrating immune and nonimmune stromal cell populations.

### Identification of diagnostic markers

To screen and identify the potential diagnostic indicators, the “randomForest” package (Izmirlian, 2004) was used to construct the prediction model. We ranked genes by the importance of variation, and the top 20 genes as well as 10 hub genes were combined as candidate diagnostic genes. The GSE28146 dataset was used to perform receiver operating characteristic (ROC) curve analysis to assess the predictive value of the candidate genes and their expression levels were compared between AD patients and control subjects.

### Correlation analysis of diagnostic markers and infiltrating immune cells

**Figure 1:**
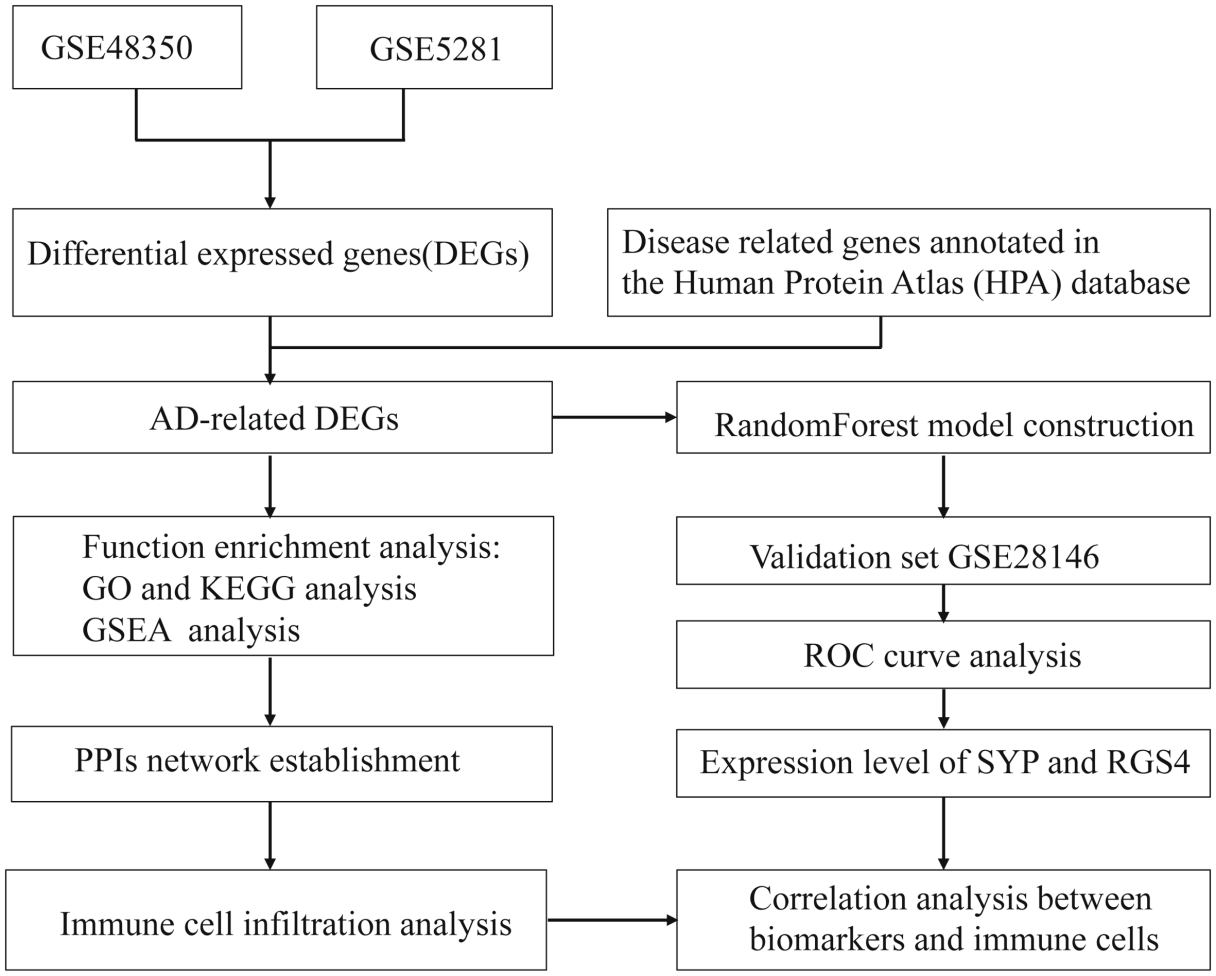
The workflow of the study.

Spearman correlation analysis was performed to assess the relationship between the expression of the identified diagnostic markers and immune cell infiltration with the results visualized using the “ggplot2” package. The workflow of the study is shown in Figure 1.

### Statistical analysis

Data preparation, functional analysis, and modeling were performed using R v4.1.1 (see text footnote 2). SPSS v15.0 software (IBM, Armonk, NY, United States) was used for statistical analyses. Data are presented as mean ± SD and the unpaired t test was used to evaluate mean differences between the 2 groups. Correlations were determined with Spearman’s analysis. Differences with *p* < 0.05 were considered statistically significant.

## Results

### Data preprocessing and differentially expressed gene identification

**Figure 2:**
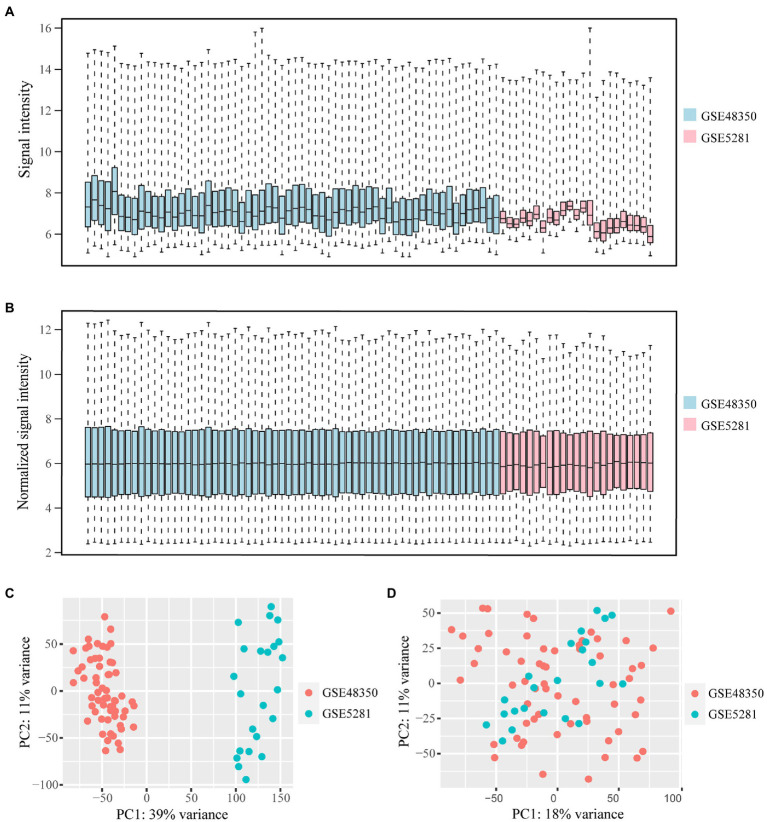
Data from GSE483501 and GSE5281 datasets is normalized and the batch effect is removed. **(A,B)** The box plot of the batch effect of GSE483501 and GSE5281 datasets before and after normalization. The blue boxes represent the signal intensity of the GSE48351 data distribution, the pink boxes represent the signal intensity of the GSE5281 data distribution. **(C)** Two-dimensional PCA cluster plot of GSE483501 and GSE5281 datasets before batch effect removal. The green dots represent samples from control group. The orange dots represent samples from AD group. **(D)** Two-dimensional PCA cluster plot of GSE483501 and GSE5281 datasets after batch effect removal.

**Figure 3:**
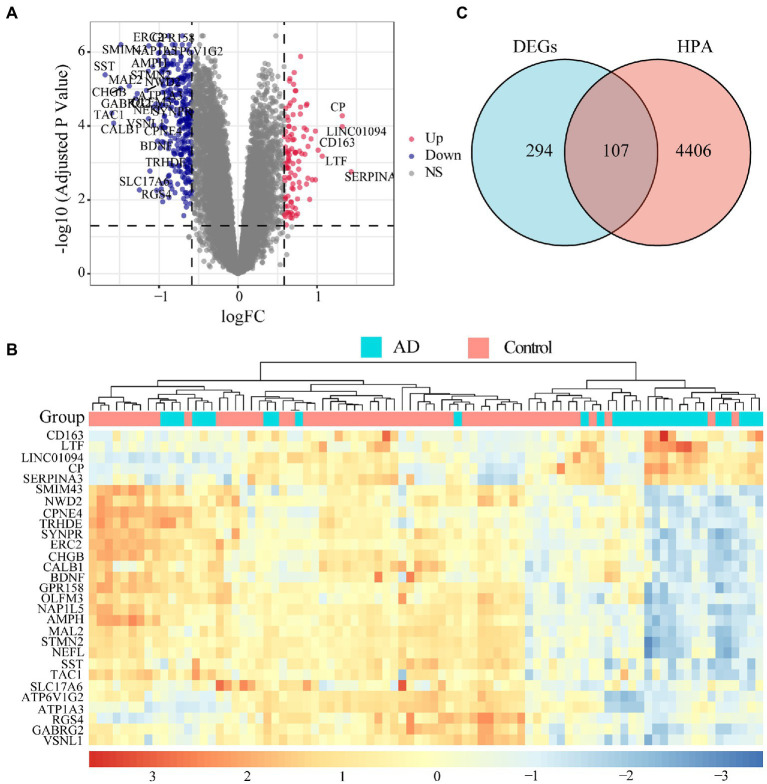
Landscape of differentially expressed genes (DEGs). **(A)** Volcano map of DEGs. Red dots represent up-regulated differential genes, blue dots represent down-regulated differential genes, and grey dots represent no significant difference genes. The DEGs with |log_2_FC| > 1 and FDR < 0.01 are marked on the map. **(B)** An expression heat map of DEGs with |log_2_FC| > 1 and FDR < 0.01. **(C)** The genes related to disease annotated in the HPA database were intersected with DEGs, 107 AD-related DEGs were screened out.

Microarray data of human hippocampus tissue from 29 AD patients and 56 normal controls were extracted from GSE48350 and GSE5281 datasets. A boxplot was used to show the batch effect (Figure 2A), and the combined gene expression matrix was normalized (Figure 2B). The two-dimensional PCA cluster diagram showed optimal clustering after eliminating the batch effect (Figures 2C,D), indicating that the sample source was reliable. In total, 401 DEGs between the AD and control groups were extracted from the gene expression matrix based on the criteria |log_2_FC| > 0.585 and FDR < 0.05 (Supplementary Table 1); the genes were visualized in a volcano map (Figure 3A). There were 104 upregulated and 297 downregulated DEGs. The heat map showed that the expression of 29 of these genes differed significantly between the two groups (|log_2_FC| > 1 and FDR < 0.01) (Figure 3B). To identify genes with diagnostic utility for AD, the DEGs were intersected with 107 AD-related genes in the HPA database (Figure 3C; Supplementary Table 2). The top 5 genes upregulated in AD compared with the control were *CP*, *CXCR4*, *PRKX*, *AEBP1*, and *GLIS3*; and the top 5 downregulated genes were *GABRG2*, *ATP1A3*, *NEFL*, *RGS4*, *BDNF*.

### Functional enrichment analyses

**Figure 4:**
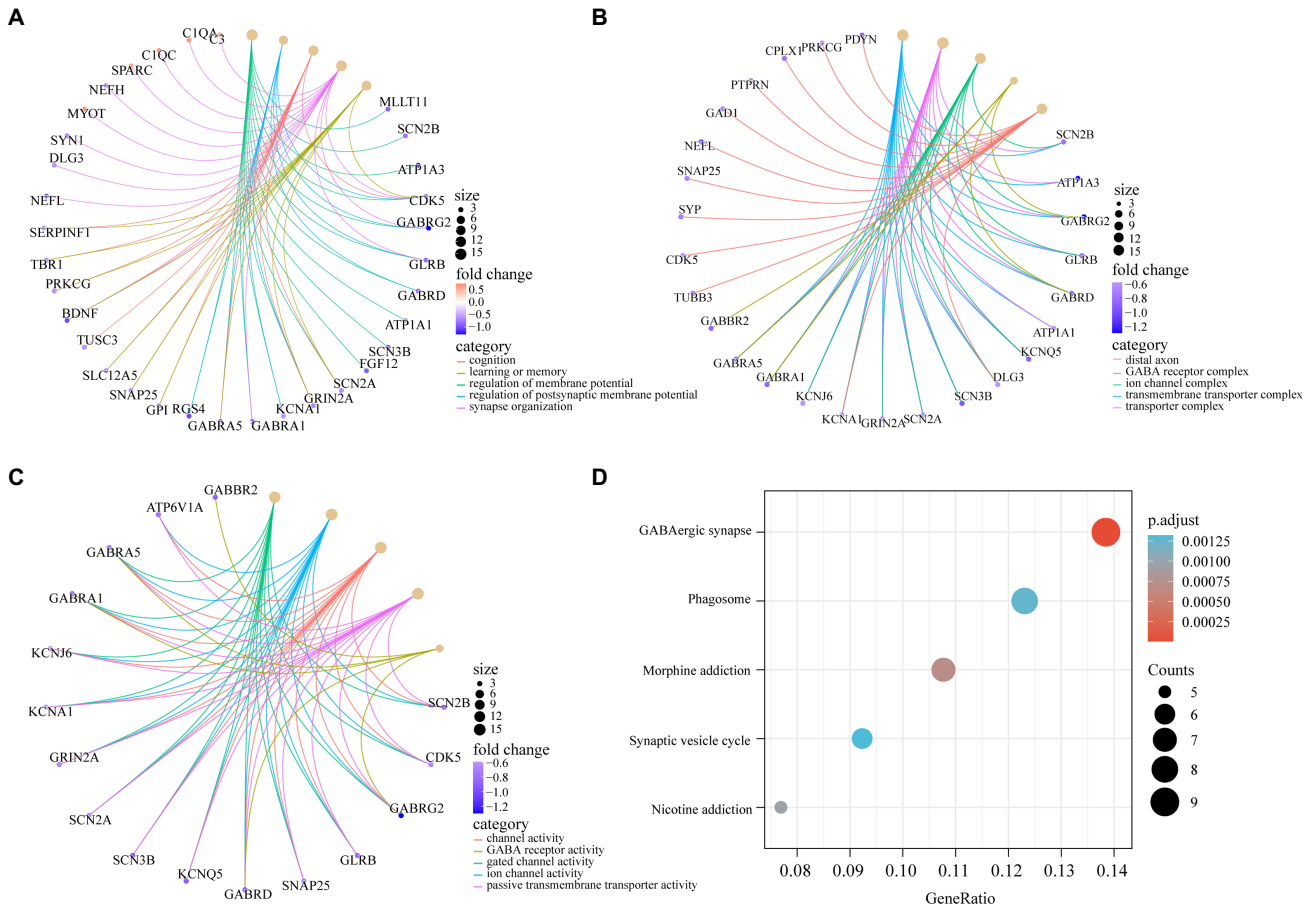
GO and KEGG pathway enrichment of AD-related DEGs. **(A)** The circle graph shows the AD-related DEGs enriched in the top5 GO categories of BPs. **(B)** The circle graph shows the AD-related DEGs enriched in the top5 GO categories of CCs. **(C)** The circle graph shows the AD-related DEGs enriched in the top5 GO categories of MFs. The yellow dots represent the GO categories, the color of the line delivered by a dot indicates the category of the dot in the legend, the size of a dot indicates the number of the genes included. **(D)** The dot plot shows the AD-related DEGs enriched in the top5 KEGG pathways.

**Figure 5:**
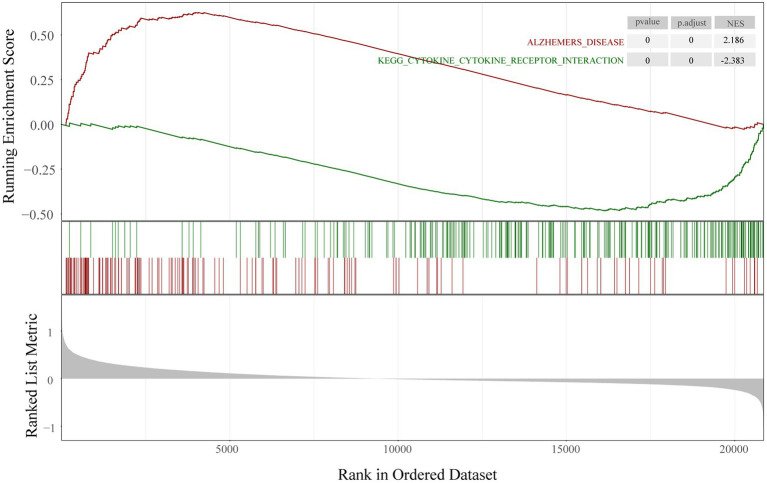
Gene set enrichment analysis. Running enrichment score was positive in Alzheimer’s disease and negative in KEGG cytokine-cytokine receptor interaction pathway.

To determine the function of the AD-related DEGs, GO, and KEGG pathway enrichment analyses were carried out. Links between proteins and the top five biological process, cellular components, or molecular function GO terms with the smallest *p* value were visualized with cnetplot. Among biological process terms, AD-related DEGs were mainly enriched in negative regulation of Cognition, Learning or memory, Synapse organization, Synaptic vesicle cycle, and Vesicle-mediated transport in synapse (Figure 4A). Among cellular component terms, the DEGs were mainly enriched in Negative regulation of exocytic vesicle membrane, Synaptic vesicle, Synaptic vesicle membrane, Transport vesicle, and Transport vesicle membrane (Figure 4B). For molecular function terms, AD-related DEGs were mainly enriched in Negative regulation of ATPase-coupled ion transmembrane transporter activity, GABA receptor activity, Inhibitory extracellular ligand-gated ion channel activity, Neurotransmitter receptor activity involved in regulation of postsynaptic membrane potential, and Structural constituent of synapse (Figure 4C). There were 11 genes enriched in all three GO categories (*SCN2B*, *CDK5*, *GABRG2*, *GLRB*, *GABRD*, *SCN3B*, *SCN2A*, *GRIN2A*, *KCNA1*, *GABRA1*, and *SNAP25*). A bubble chart was used to match the AD-related DEGs with the top 5 KEGG pathways; the genes were enriched in GABAergic synapse, morphine addiction, nicotine addiction, phagosome, and synaptic vesicle cycle (Figure 4D). *KCNJ6*, *PRKCG*, *GABRG2*, *ATP6V1A*, *ATP6V1B2*, *ATP6V1E1*, *GABRA1*, *GABRA5*, and *GABRD* were enriched in at least 2 KEGG pathways. GSEA showed that the enriched genes had an AD running enrichment score of 2.186 and KEGG cytokine-cytokine receptor interaction running enrichment score of −2.383 (Figure 5).

### Protein–protein interaction network establishment and hub gene identification

**Figure 6:**
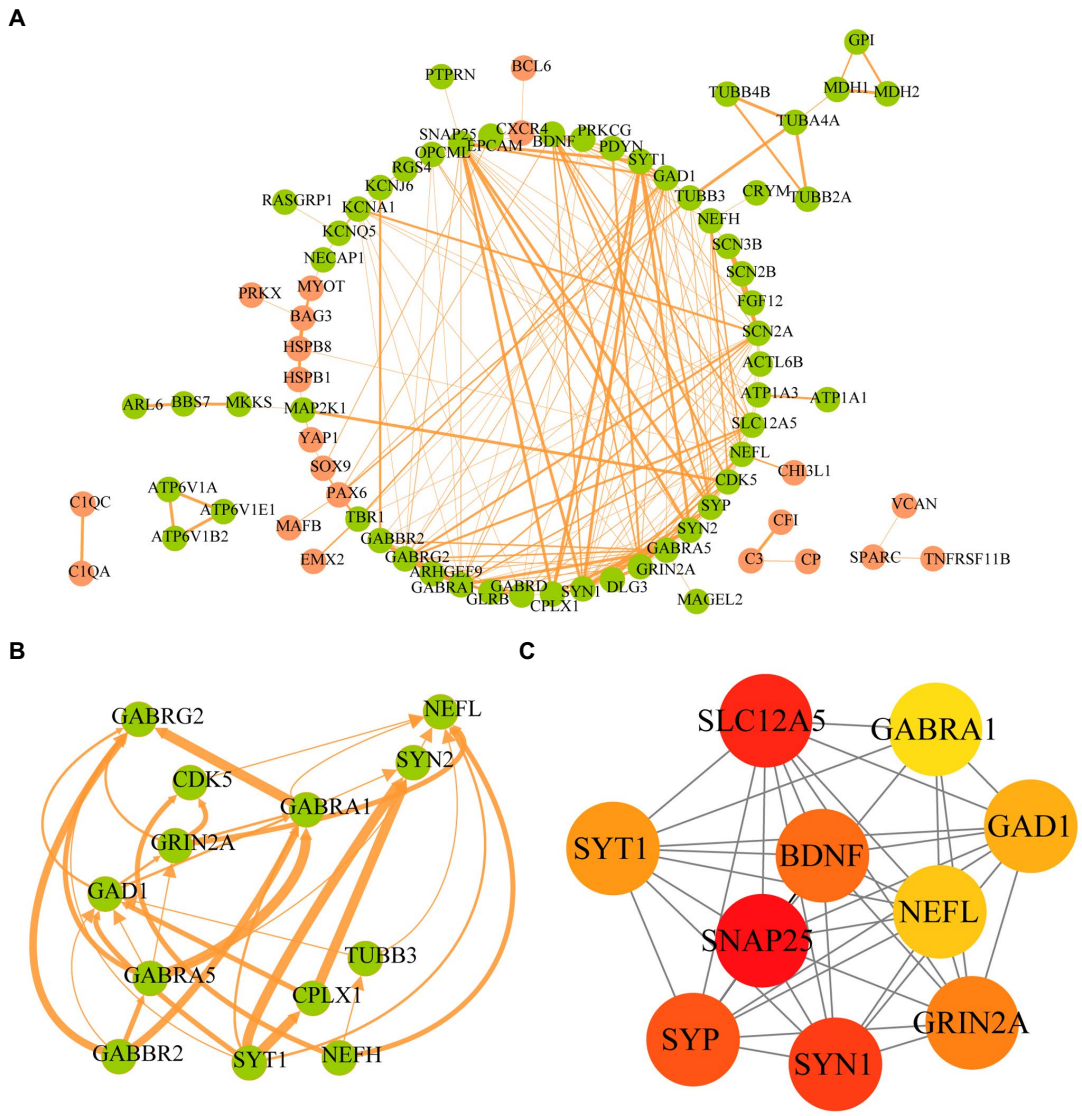
Protein–protein interaction network of AD-related DEGs and screening of hub genes. **(A)** The STRING database is used to construct the PPI network of AD-related, with 77 nodes and 198 edges. The upregulated genes are shown in orange and the downregulated genes are shown in green. The wider the edge, the higher the combined score. **(B)** The gene cluster with the highest score of AD-related DEGs PPI network. **(C)** The top10 hub genes constructed by the MCC method using Cytohubba plugin.

To clarify the interactions between the proteins corresponding to the AD-related DEGs, the STRING database was used to construct a PPI network of 107 disease-related DEGs (Figure 6A). The network comprised 77 nodes and 198 edges. We used the MCODE plugin to construct functional clusters; 2 clusters had an MCODE score > 5. Cluster 1 contained 13 nodes and 34 edges (Figure 6B), whereas cluster 2 contained 7 nodes and 12 edges (data not shown). The cytoHubba plugin and maximal clique centrality (MCC), edge percolated component, and degree methods were used to screen and identify the top 10 hub genes (Supplementary Table 3). The top 10 hub genes ranked with the MCC method were *SNAP25*, *SLC12A5*, *SYN1*, *SYP*, *BDNF*, *GRIN2A*, *SYT1*, *GAD1*, *NEFL*, and *GABRA1* (Figure 6C); *GRIN2A* and *NEFL* were also present in the functional gene modules constructed with MCODE.

### Immune cell infiltration analysis

**Figure 7:**
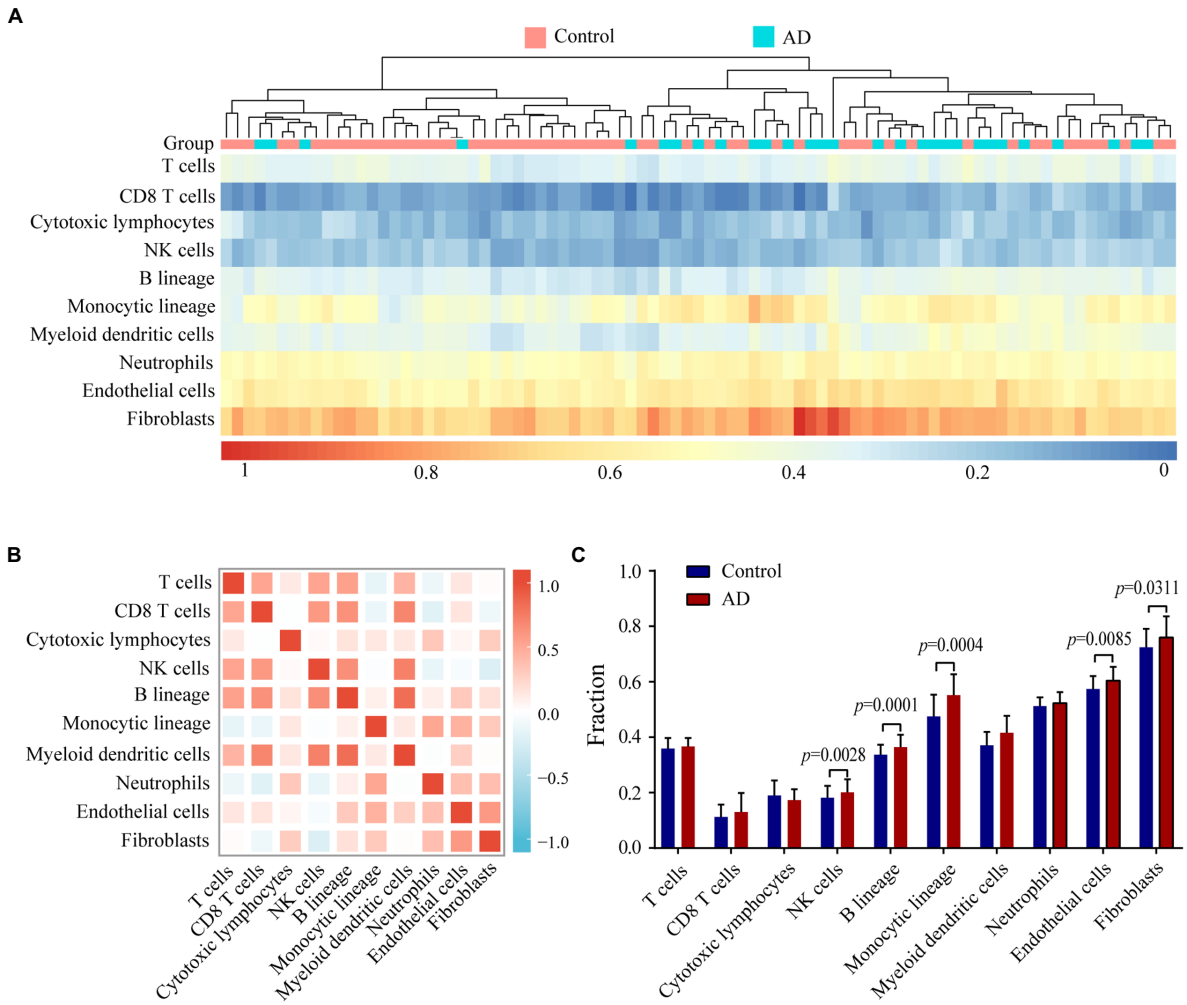
Evaluating and identifying the AD immune microenvironment. **(A)** Heat map presenting results of 8 types of immune cells and 2 types of non-immune stromal cells. **(B)** Correlations heat map of 8 types of immune cells and 2 types of non-immune stromal cells. Red represents a negative correlation, and blue represents a positive correlation. The darker the color, the stronger the correlation. **(C)** Bar plot illustrating the proportion of 8 types of immune cells and 2 types of non-immune stromal cells between AD (red) and control (blue) group.

To evaluate immune status in AD patients, infiltrating cells were analyzed with the MCP-counter algorithm. The heat map shows the distribution of 8 types of infiltrating immune cell and 2 types of stromal cell (Figure 7A). The correlation heat map of the 10 cell types revealed a significant positive correlation between CD8^+^ T cell, NK cell, B lineage cell, and myeloid dendritic cell numbers. Monocytic lineage cell, neutrophil, and endothelial cell numbers were positively correlated whereas NK cell numbers showed a significant negative correlation with the number of fibroblasts (Figure 7B). AD patients showed higher numbers of NK cells, B lineage cells, monocytic lineage cells, endothelial cells, and fibroblasts compared with healthy controls (Figure 7C).

### Screening and verification of diagnostic markers

**Figure 8:**
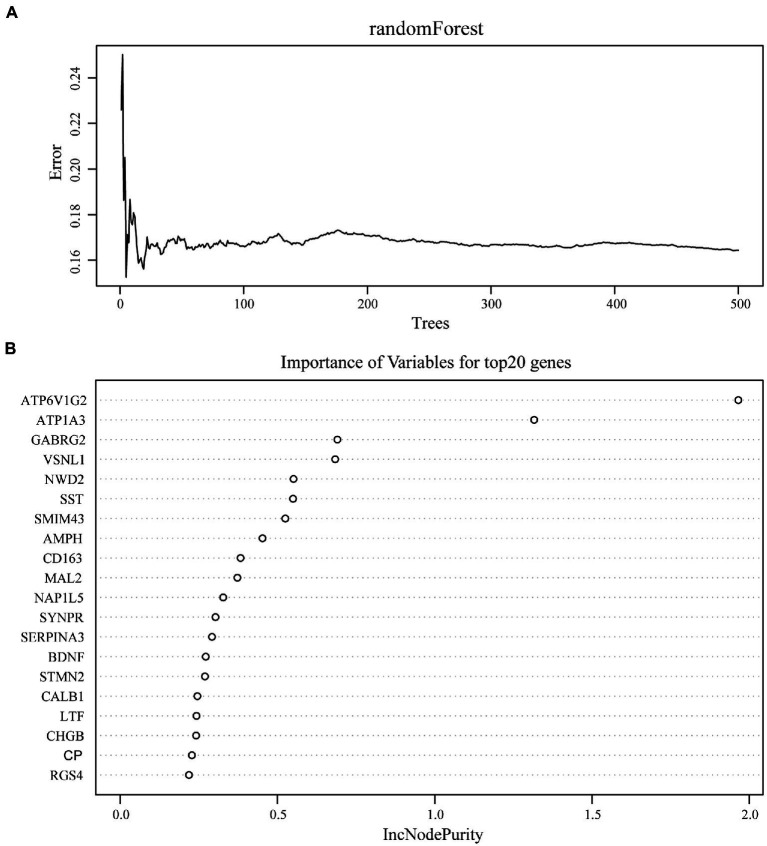
Screening of diagnostic candidates by machine-learning approach. **(A)** The randomForest algorithm is used to construct a prediction model. **(B)** Importance of variables for top20 genes recognized from random forest analysis.

**Figure 9:**
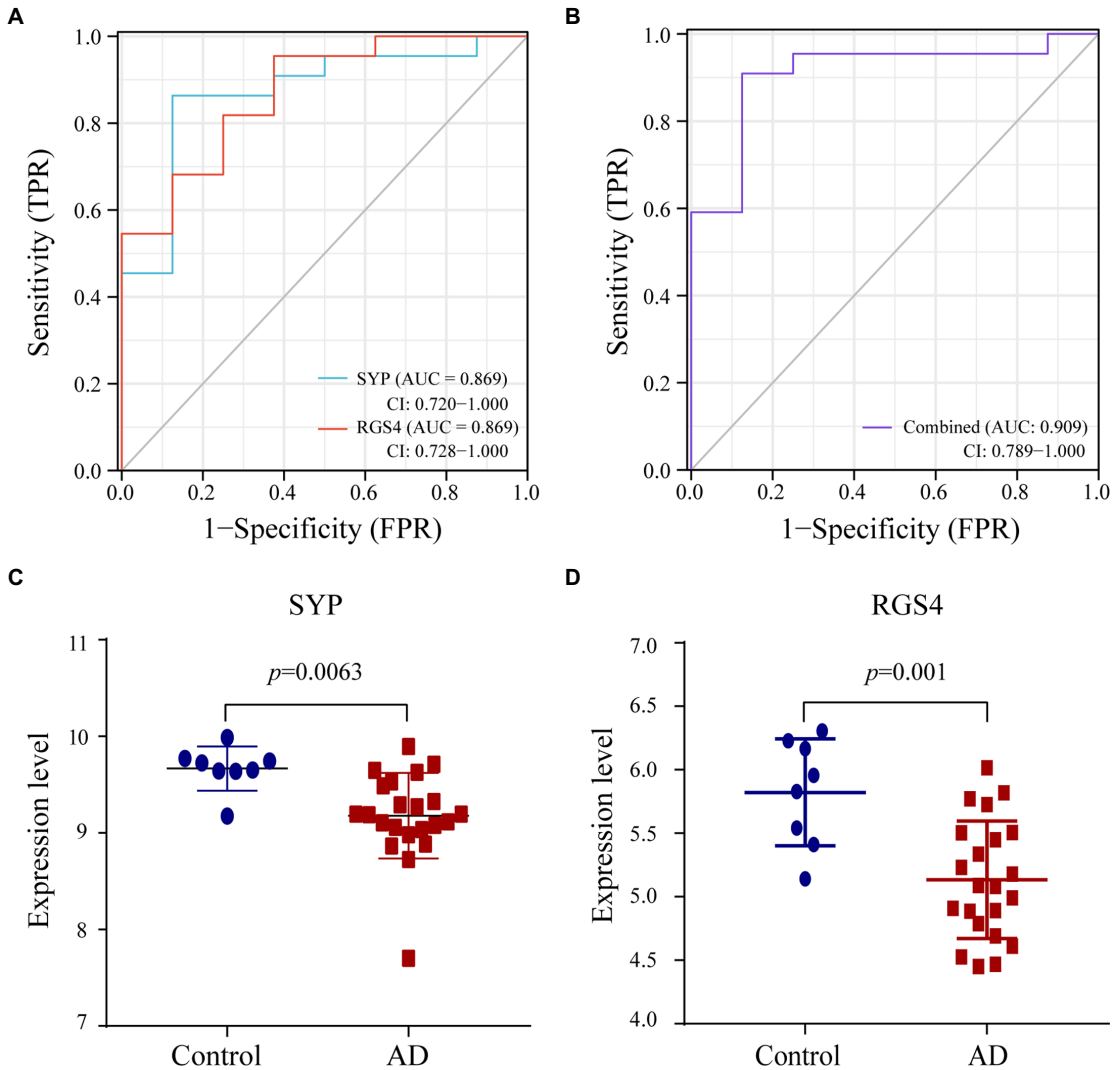
Verification of diagnostic markers. **(A,B)** The ROC curve of the diagnostic efficacy in verification set GSE28146. **(C,D)** The expression level of SYP and RGS4 in hippocampus between AD and control group from verification set.

We used the random Forest algorithm to construct a prediction model (Figure 8A) and found significant differences in predictive scores for diagnosis using training and test datasets (Supplementary Figure 1). After calculating the importance of variation, the top 20 genes were selected as candidate markers for AD diagnosis (Figure 8B; Supplementary Table 4). To evaluate the predictive utility of these markers, ROC curve analysis was performed for the diagnostic model using the GSE28146 dataset (Supplementary Tables 5, 6). *RGS4* had the highest area under the ROC curve (AUC; 0.869) among the top 20 candidate marker genes, whereas *SYP* had the highest AUC (0.869) among the 10 hub genes (Figure 9A). When the 2 genes were combined into a single variable, the predictive value improved in the validation set (AUC = 0.909, 95% confidence interval: 0.789–1.000; Figure 9B). Additionally, *RGS4* and *SYP* were downregulated in the hippocampus of AD patients compared with healthy controls in the validation set (Figures 9C,D). These results suggest that *RGS4* and *SYP* have diagnostic value for predicting AD with high accuracy.

### Correlation analysis between diagnostic markers and immune cells

**Figure 10:**
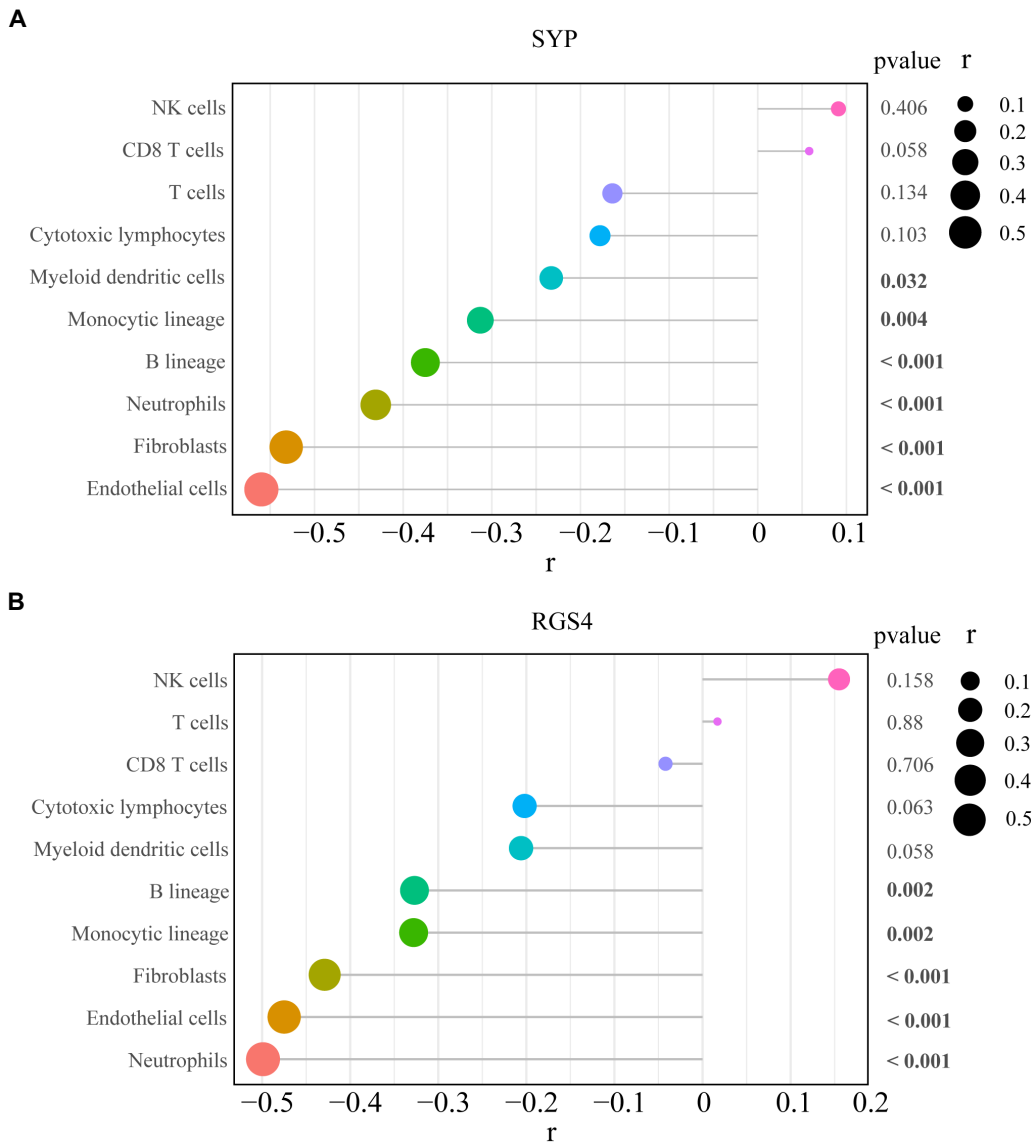
Correlation between diagnostic markers and 8 types of immune cells and 2 types of non-immune stromal cells. **(A)** Correlation between SYP and 10 types of cells. **(B)** Correlation between RGS4 and 10 types of cells. The larger dots represent the stronger correlation between genes and cells. *p* < 0.05 indicated statistically significant.

The correlation analysis showed that *SYP* expression was negatively correlated with the number of monocytic lineage cells (*r* = −0.313, *p* = 0.004), myeloid dendritic cells (*r* = −0.233, *p* = 0.032), B lineage cells (*r* = −0.375, *p* < 0.001), neutrophils (*r* = −0.431, *p* < 0.001), endothelial cells (*r* = −0.56, *p* < 0.001), and fibroblasts (*r* = −0.532, *p* = 0.031; Figure 10A). Meanwhile, *RGS4* expression was negatively correlated with the number of monocytic lineage cells (*r* = −0.328, *p* = 0.002), B lineage cells (*r* = −0.327, *p* = 0.002), neutrophils (*r* = −0.499, *p* < 0.001), endothelial cells (*r* = −0.475, *p* < 0.001), and fibroblasts (*r* = −0.429, *p* < 0.001; Figure 10B).

## Discussion

Alzheimer disease is primarily diagnosed based on pathologic Aβ deposition and tau (total and phosphorylated tau) accumulation (DeTure and Dickson, 2019). The mechanisms underlying the progressive accumulation of Aβ and neurofibrillary tangles in AD remain unknown. Immune cells participate in neuroinflammation and Aβ clearance, and potentially contribute to the neuropathology of AD (Britschgi et al., 2009; Baulch et al., 2020). It is therefore important to identify novel molecular markers for the early diagnosis of AD and clarify the role of immune cells in AD pathogenesis. In this study, we identified *SYP* and *RGS4* as novel diagnostic markers for AD, and found that myeloid cells may participate in neuroinflammation and thereby contribute to synapse loss in the pathogenesis of this disease.

We integrated and analyzed the GSE48350 and GSE5281 datasets and identified 107 AD-related DEGs. GO enrichment showed that AD-related DEGs were mainly enriched in the terms Regulation of membrane potential, Regulation of postsynaptic membrane potential, Cognition, Synapse organization, and Learning or memory. *GABRG2*, *GABRD*, *RGS4*, *GABRA1*, *GPI*, *SNAP25*, *SLC12A5*, *PRKCG*, *TBR1*, *SERPINF1*, *GLRB*, *SCN2A*, *BDNF*, *CDK5*, *GRIN2A*, and *GABRA5* were enriched in at least 2 of these 5 biological processes, implying that these genes impact the structure and function of synapses in AD development. KEGG pathway enrichment analysis showed that upregulated AD-related DEGs were mainly enriched in complement and coagulation cascades, whereas downregulated AD-related DEGs were mainly enriched in GABAergic synapse. The complement system is essential for the innate and adaptive immune responses, which inhibit the formation and promote the clearance of amyloid plaques (Morgan, 2018). Dysregulation of the complement cascade can lead to chronic neuroinflammation and neurodegeneration associated with AD (Eikelenboom and Veerhuis, 1996; Shen and Meri, 2003). Complement cooperates with microglia in synapse elimination, resulting in hippocampal dysfunction (Hong et al., 2016). The complement and coagulation cascades are significantly altered in both the peripheral blood and central nervous system (CNS) of patients with AD (Chen and Xia, 2020). Thus, complement system activation may be related to AD pathology. It was also reported that dysfunctional GABAergic neurons contribute to amyloidosis and tauopathy, causing altered synaptic plasticity and severe memory impairment (Loreth et al., 2012; Levenga et al., 2013; Soler et al., 2017). By analyzing the PPI network of AD-related DEGs, we identified *SNAP25*, *SLC12A5*, *SYN1*, *GRIN2A*, *GAD1*, *NEFL*, and *GABRA1* as being among the top 10 hub genes screened using 3 different topologic methods. Synaptosomal-associated protein 25 kDa (*SNAP25*) was the top gene with all 3 methods. SNAP25 is a calcium-dependent protein that is expressed at the presynaptic terminals of mature GABAergic neurons and is a component of the SNARE complex that stimulates GABA release (Tafoya et al., 2006). Our results suggest that GABAergic signaling is an important mechanism of AD pathogenesis and progression.

We used a machine learning approach to construct a diagnostic model and identified the top 20 genes as candidate markers for AD diagnosis. Combined with the top 10 AD-related hub genes, a total of 30 putative markers were validated in validation set, including synaptophysin (*SYP*) and regulator of G protein signaling 4 (*RGS4*). SYP is an integral membrane protein of synaptic vesicles and a marker of synaptic density and synaptogenesis (Evans and Cousin, 2005). SYP is associated with neuronal plasticity in learning and memory (Fagnou and Tuchek, 1995); aberrant SYP expression has been linked to synaptic loss in neurodegenerative diseases including AD and Parkinson disease (PD; Zhan et al., 1993; Dinda et al., 2019). RGS4 is a small regulator of the 23-kDa G protein that is abundantly expressed in the CNS and heart in humans (Zhang et al., 1998; Erdely et al., 2004). In the CNS, *RGS4* mRNA is present at high levels in the amygdala and striatum but is also detected in most cortical layers (Ebert et al., 2006). As a GTPase-activating protein for Gαq and Gαi/o, RGS4 provides negative feedback regulation of postsynaptic G protein-coupled signaling pathways in synapse assembly (Fossella et al., 2003). *RGS4* is known to be a schizophrenia susceptibility gene and is downregulated in the cortex of schizophrenia patients (Schwarz, 2018). In the striatum, RGS4 regulates cholinergic and dopaminergic signaling; its dysregulation is associated with motor deficits in PD (Geurts et al., 2003; Ding et al., 2006). Given its role in synaptogenesis, RGS4 may be involved in synaptic loss in AD and consequent cognitive defects.

Neuroinflammation contributes to AD progression by altering the expression of neuronal synaptic proteins. AD patients show a high infiltration of NK cells, B lineage cells, monocytic lineage cells, endothelial cells, and fibroblasts. NK cells have been implicated in neuroinflammation in AD patients and are involved in the pathogenesis of the disease (Krishnaraj, 1991; Camous et al., 2012). Genomic analyses have identified AD risk genes that modulate the epigenome and transcriptome of myeloid lineage cells including monocytes, macrophages, and microglia (Huang et al., 2017; Finucane et al., 2018) as well as their enhancers (Novikova et al., 2021). Although there is limited evidence of a pathogenic or protective role of B cells in AD, B cell depletion has been shown to delay AD progression in mice by reducing Aβ plaque accumulation and inhibiting disease-associated microglia (Kim et al., 2021). Endothelial cells and fibroblasts are the main cell types that constitute blood–brain barrier (BBB). Neurovascular dysfunction and BBB breakdown have been observed in the hippocampus during aging (Montagne et al., 2015). Thus, NK cells, B lineage cells, monocytic lineage cells, endothelial cells, and fibroblasts may be components of the immune microenvironment in AD. A correlation analysis showed that *RGS4* and *SYP* expression was negatively correlated with the number of monocytic lineage cells, B lineage cells, neutrophils, endothelial cells, and fibroblasts; there was also a negative correlation between *SYP* expression and myeloid dendritic cell numbers. These results indicate that myeloid cells may be involved in neuroinflammation-related synaptic loss in the hippocampus of AD patients. As few is known about SYP and RGS4 function in the immune response, their specific physiology in regulating immune microenvironment of AD is worthy of further study and exploration. To summarize, we found that SYP and RGS4 are potential biomarkers of AD, and may affect the pathogenesis of AD by influencing immune microenvironment.

## Conclusion

The study revealed that *SYP* and *RGS4* is related to the AD pathological process, and could be potential diagnostic markers of AD. Besides, it is noteworthy that the close relationship between immune microenvironment and AD neuroinflammation in the pathological process of AD. These findings may shed light on potential early diagnosis and novel immunotherapy of AD.

## Data availability statement

The original contributions presented in the study are included in the article/supplementary material, further inquiries can be directed to the corresponding authors.

## Author contributions

DC, BL, and JZ conceived and designed the study. RQ, XK, HZ, and XW contributed to data collection. DC, YZ, BL, and JZ analyzed the data and drafted the manuscript. All authors contributed to the article and approved the submitted version.

## Funding

This work was supported by the National Natural Science Foundation of China (grant number: 82174522).

## Conflict of interest

The authors declare that the research was conducted in the absence of any commercial or financial relationships that could be construed as a potential conflict of interest.

## Publisher’s note

All claims expressed in this article are solely those of the authors and do not necessarily represent those of their affiliated organizations, or those of the publisher, the editors and the reviewers. Any product that may be evaluated in this article, or claim that may be made by its manufacturer, is not guaranteed or endorsed by the publisher.

## Footnotes

^1^ http://www.ncbi.nlm.nih.gov/geo

^2^ http://www.r-project.org

^3^ http://string-db.org

^4^ https://cran.r-project.org/web/packages/pheatmap/index.html
