# Supplementary material for: Integrated bioinformatics-based identification of diagnostic markers in Alzheimer disease
Source: Front Aging Neurosci. 2022 Nov 10;14:988143. doi: 10.3389/fnagi.2022.988143 (PMC9686423; doi:10.3389/fnagi.2022.988143)
Supplement: Supplementary file 1 [file Data_Sheet_1.docx]

Supplementary Material

## Supplementary Tables

Table 1. 401 DEGs with |log_2_FC|>0.585 and adjusted *p*-value (FDR) <0.05

| Symbol | Log_2_FC | AveExpression | adj.P.Val |
| --- | --- | --- | --- |
| NRXN3 | -0.87973 | 8.568903 | 3.64E-07 |
| CISD1 | -0.7044 | 9.521354 | 3.64E-07 |
| TUBB2A | -0.70748 | 12.49273 | 6.31E-07 |
| TUBB3 | -0.79728 | 11.19398 | 6.31E-07 |
| SCG5 | -0.83118 | 11.09957 | 6.31E-07 |
| SMIM43 | -1.48451 | 7.41347 | 6.31E-07 |
| GPR158 | -1.00152 | 9.538347 | 6.31E-07 |
| ATP6V1E1 | -0.62018 | 10.9412 | 6.31E-07 |
| COPG2IT1 | -0.98006 | 9.775181 | 6.51E-07 |
| ERC2 | -1.13106 | 9.434816 | 6.82E-07 |
| NUP93 | -0.64254 | 7.496113 | 8.72E-07 |
| MKKS | -0.58951 | 8.126865 | 1.02E-06 |
| RNF175 | -0.82095 | 8.485582 | 1.02E-06 |
| MLLT11 | -0.88569 | 11.8331 | 1.02E-06 |
| TUBA4A | -0.99256 | 10.11261 | 1.02E-06 |
| NAP1L3 | -0.7606 | 10.71221 | 1.04E-06 |
| NAP1L5 | -1.01082 | 10.30386 | 1.08E-06 |
| CALY | -0.72039 | 7.762141 | 1.32E-06 |
| WWTR1 | 0.791854 | 7.091331 | 1.32E-06 |
| SNCB | -0.93401 | 8.367672 | 1.32E-06 |
| MTX2 | -0.72444 | 8.338533 | 1.32E-06 |
| CA10 | -0.90658 | 7.876458 | 1.66E-06 |
| ATP6V1G2 | -1.00912 | 10.21963 | 1.66E-06 |
| TUBB4B | -0.61774 | 11.17988 | 1.66E-06 |
| ENO2 | -0.72626 | 10.93676 | 1.71E-06 |
| FABP3 | -0.81816 | 7.129757 | 1.71E-06 |
| SLC1A6 | -0.8518 | 5.909644 | 2.02E-06 |
| SNAP91 | -0.97257 | 10.37474 | 2.05E-06 |
| MANEAL | -0.58512 | 7.612562 | 2.05E-06 |
| MDH1 | -0.88424 | 11.8139 | 2.08E-06 |
| ADAM23 | -0.59584 | 6.725964 | 2.08E-06 |
| ACTR10 | -0.64687 | 9.908504 | 2.09E-06 |
| ELOVL4 | -0.84095 | 7.77513 | 2.16E-06 |
| NECAP1 | -0.88778 | 9.546775 | 2.16E-06 |
| MEST | -0.7708 | 8.861801 | 2.17E-06 |
| PNMA8A | -0.8198 | 10.51364 | 2.17E-06 |
| ACOT7 | -0.90109 | 8.661075 | 2.41E-06 |
| GPI | -0.6639 | 10.50407 | 2.41E-06 |
| MDH2 | -0.61265 | 9.115255 | 2.41E-06 |
| AMPH | -1.07991 | 9.511152 | 2.49E-06 |
| PSMA5 | -0.66119 | 8.227758 | 2.88E-06 |
| SH3GL2 | -0.92354 | 10.40756 | 2.91E-06 |
| ATP8A2 | -0.75785 | 8.252351 | 2.91E-06 |
| BEX5 | -0.95235 | 10.43759 | 2.91E-06 |
| STMN2 | -1.13793 | 11.13874 | 3.29E-06 |
| PKIB | -0.96812 | 5.096267 | 3.30E-06 |
| HMGCR | -0.6873 | 8.662496 | 3.53E-06 |
| GOT1 | -0.96258 | 9.245564 | 3.56E-06 |
| S1PR3 | 0.709455 | 6.692255 | 3.58E-06 |
| SVOP | -0.87302 | 7.72893 | 3.59E-06 |
| SST | -1.68356 | 8.220447 | 4.11E-06 |
| ATP5F1B | -0.59694 | 11.32274 | 4.11E-06 |
| UBE2V2 | -0.64125 | 7.406286 | 4.19E-06 |
| FAM216A | -0.66897 | 7.775761 | 4.32E-06 |
| NETO1 | -0.63712 | 6.424845 | 4.56E-06 |
| NDRG4 | -0.71709 | 11.31973 | 4.68E-06 |
| MOAP1 | -0.72757 | 10.81928 | 5.01E-06 |
| TSPAN13 | -0.69292 | 10.0694 | 5.01E-06 |
| SYT13 | -0.89962 | 8.800074 | 5.01E-06 |
| FAM102B | -0.74954 | 8.297069 | 5.07E-06 |
| PGAP4 | -0.721 | 8.917354 | 5.19E-06 |
| MRPL15 | -0.69991 | 8.283668 | 5.22E-06 |
| TLR5 | 0.655057 | 6.81517 | 5.22E-06 |
| BBS7 | -0.69337 | 6.932926 | 5.27E-06 |
| TSPYL5 | -0.61008 | 8.5995 | 5.59E-06 |
| PSMG1 | -0.77889 | 6.454698 | 5.63E-06 |
| MAP7D2 | -0.81779 | 8.640452 | 5.68E-06 |
| SYT4 | -0.83978 | 9.809458 | 5.72E-06 |
| BEX1 | -0.67252 | 12.29964 | 5.72E-06 |
| GNG3 | -0.94473 | 9.579008 | 5.88E-06 |
| C1QTNF4 | -0.87389 | 7.567921 | 5.88E-06 |
| TNFRSF11B | 0.65058 | 4.0091 | 6.05E-06 |
| NSG2 | -0.80908 | 10.02981 | 6.10E-06 |
| JPT1 | -0.69937 | 8.035358 | 6.55E-06 |
| SCN2B | -0.76818 | 7.762376 | 6.55E-06 |
| CLSTN2 | -0.8494 | 8.465196 | 7.15E-06 |
| ANGPT1 | 0.703111 | 5.540001 | 7.42E-06 |
| INA | -0.95427 | 9.821803 | 7.43E-06 |
| REEP1 | -0.76418 | 9.589363 | 7.43E-06 |
| NME1 | -0.75331 | 9.792186 | 7.48E-06 |
| EEF1A2 | -0.86877 | 10.72831 | 7.50E-06 |
| SLC16A14 | -0.61945 | 8.238307 | 7.60E-06 |
| MAL2 | -1.37794 | 8.464939 | 8.28E-06 |
| SYN2 | -0.94095 | 7.61468 | 8.30E-06 |
| SIDT1 | -0.66494 | 7.602437 | 9.32E-06 |
| RAB3C | -0.78676 | 6.282702 | 9.47E-06 |
| CHGB | -1.48771 | 8.861323 | 9.67E-06 |
| GABBR2 | -0.85482 | 9.388268 | 1.03E-05 |
| NWD2 | -1.16039 | 6.027666 | 1.09E-05 |
| KIFAP3 | -0.88769 | 9.745906 | 1.09E-05 |
| MAGEL2 | -0.62569 | 7.265276 | 1.10E-05 |
| CYP4X1 | -0.74276 | 7.271122 | 1.10E-05 |
| PLPPR5 | -0.72837 | 6.960534 | 1.11E-05 |
| VCAN | 0.724317 | 8.094979 | 1.11E-05 |
| S100A4 | 0.729713 | 6.513947 | 1.11E-05 |
| HENMT1 | -0.60335 | 7.420996 | 1.15E-05 |
| SGIP1 | -0.66082 | 8.372689 | 1.19E-05 |
| PWWP3B | -0.93311 | 7.606845 | 1.21E-05 |
| TAFA2 | -0.95739 | 8.505471 | 1.23E-05 |
| CADPS | -0.74964 | 7.623702 | 1.26E-05 |
| NSG1 | -0.87663 | 7.844391 | 1.26E-05 |
| ATP1A3 | -1.27774 | 8.836432 | 1.32E-05 |
| CDK5 | -0.66809 | 8.960037 | 1.35E-05 |
| PDE1A | -0.8341 | 7.19282 | 1.37E-05 |
| DHRS11 | -0.61244 | 7.262972 | 1.37E-05 |
| GHITM | -0.58784 | 10.02726 | 1.41E-05 |
| BSN | -0.79572 | 9.262082 | 1.44E-05 |
| KRT222 | -0.78208 | 6.891861 | 1.44E-05 |
| SYP | -0.7292 | 8.726456 | 1.45E-05 |
| ATP6V1B2 | -0.86639 | 10.92996 | 1.46E-05 |
| GABRG2 | -1.29834 | 8.5616 | 1.77E-05 |
| TRAPPC6B | -0.69308 | 7.847749 | 1.77E-05 |
| FHL2 | -0.88737 | 8.038876 | 1.84E-05 |
| UBE2T | -0.6675 | 6.254891 | 1.88E-05 |
| LINC01616 | -0.90773 | 7.404947 | 1.89E-05 |
| RAB13 | 0.7069 | 9.12395 | 2.00E-05 |
| GLRB | -0.84464 | 7.995211 | 2.02E-05 |
| NXPH1 | -0.82069 | 6.386578 | 2.08E-05 |
| NKIRAS1 | -0.58678 | 8.616993 | 2.20E-05 |
| SNAP25 | -0.69873 | 10.80515 | 2.23E-05 |
| SLIT1 | -0.69633 | 8.335111 | 2.23E-05 |
| SLC25A12 | -0.58867 | 8.555733 | 2.33E-05 |
| NUDT11 | -0.78227 | 8.250392 | 2.37E-05 |
| RASGRP1 | -0.77887 | 8.917278 | 2.37E-05 |
| NOTCH2NLA | 0.661636 | 9.128583 | 2.40E-05 |
| GPRASP1 | -0.60243 | 10.13732 | 2.47E-05 |
| OLFM3 | -1.03923 | 7.059498 | 2.52E-05 |
| PLSCR4 | 0.872048 | 8.979374 | 2.54E-05 |
| PNMA8B | -0.64291 | 9.020447 | 2.58E-05 |
| SYT1 | -0.97447 | 11.05236 | 2.65E-05 |
| SLC7A14 | -0.69939 | 6.988239 | 2.66E-05 |
| GABRD | -0.91059 | 7.321332 | 2.72E-05 |
| PPP1R14C | -0.66398 | 7.289663 | 2.74E-05 |
| PTPRR | -0.95438 | 7.033932 | 2.76E-05 |
| PRKX | 0.863381 | 6.706232 | 2.76E-05 |
| MS4A14 | 0.646145 | 5.501482 | 2.84E-05 |
| SLC12A5 | -0.68313 | 10.34278 | 2.95E-05 |
| NEFL | -1.07388 | 9.783472 | 3.07E-05 |
| SNX10 | -0.84203 | 8.588832 | 3.09E-05 |
| TGFBR3 | 0.789962 | 7.760894 | 3.09E-05 |
| SYNPR | -1.01495 | 9.897048 | 3.30E-05 |
| SRD5A1 | -0.71844 | 7.301502 | 3.32E-05 |
| SERPINI2 | 0.602895 | 5.369441 | 3.32E-05 |
| GAP43 | -0.86049 | 8.793358 | 3.34E-05 |
| NAP1L2 | -0.66953 | 9.913632 | 3.69E-05 |
| UNC13C | -0.78202 | 5.977789 | 3.69E-05 |
| OPCML | -0.62286 | 9.608385 | 4.11E-05 |
| PHF24 | -0.64505 | 8.193392 | 4.11E-05 |
| PCMT1 | -0.5982 | 9.887042 | 4.11E-05 |
| TNFRSF21 | -0.59189 | 8.220591 | 4.25E-05 |
| DCTN1-AS1 | -0.58502 | 6.615532 | 4.34E-05 |
| TAC1 | -1.59876 | 7.627698 | 4.45E-05 |
| GABRA4 | -0.64898 | 7.314416 | 4.61E-05 |
| SYT5 | -0.74255 | 7.123347 | 4.66E-05 |
| ZNF204P | -0.73436 | 7.505419 | 4.72E-05 |
| GLIS3 | 0.787527 | 6.73598 | 4.75E-05 |
| ARL6 | -0.59908 | 6.179431 | 4.79E-05 |
| CAPS | 0.696004 | 7.170896 | 4.94E-05 |
| EID2B | -0.6299 | 6.383393 | 4.98E-05 |
| ATP6V1A | -0.77958 | 9.719419 | 5.19E-05 |
| PPFIA4 | -0.61906 | 8.410052 | 5.20E-05 |
| ELAVL2 | -0.92918 | 8.3336 | 5.29E-05 |
| CP | 1.318213 | 6.839914 | 5.29E-05 |
| SMIM10L2A | -0.63004 | 8.077884 | 5.37E-05 |
| MRAP2 | -0.93491 | 7.827956 | 5.62E-05 |
| DTNA | 0.625006 | 8.838374 | 5.73E-05 |
| ATP1A1 | -0.6135 | 10.64921 | 5.94E-05 |
| NBEA | -0.6321 | 9.207437 | 6.21E-05 |
| TAGLN3 | -0.71275 | 10.59751 | 6.25E-05 |
| CD200 | -0.62629 | 8.079419 | 6.28E-05 |
| GAD1 | -0.66867 | 6.886129 | 6.35E-05 |
| VSNL1 | -1.13405 | 10.54251 | 6.35E-05 |
| RALYL | -0.66464 | 9.169842 | 6.35E-05 |
| TCEAL7 | -0.61924 | 9.801547 | 6.90E-05 |
| XK | -0.69012 | 6.86747 | 7.00E-05 |
| BCL6 | 0.634982 | 7.929246 | 7.01E-05 |
| ATRNL1 | -0.65757 | 7.11595 | 7.03E-05 |
| RBFOX1 | -0.7136 | 10.20471 | 7.14E-05 |
| NDRG3 | -0.59921 | 9.209151 | 7.14E-05 |
| SLC39A10 | -0.73674 | 9.446651 | 7.41E-05 |
| KCNQ5 | -0.90961 | 6.115755 | 7.41E-05 |
| MIR7-3HG | -0.74575 | 5.86906 | 7.64E-05 |
| XKR4 | -0.65128 | 7.392471 | 7.83E-05 |
| EPDR1 | -0.73183 | 10.25488 | 7.91E-05 |
| CALB1 | -1.57597 | 7.156687 | 8.40E-05 |
| ACTL6B | -0.65135 | 7.566793 | 8.94E-05 |
| OR2L13 | -0.74962 | 5.11406 | 8.99E-05 |
| PTPRN | -0.64578 | 7.976715 | 9.01E-05 |
| ARHGDIG | -0.59827 | 8.251121 | 9.26E-05 |
| TUSC3 | -0.62169 | 6.426824 | 9.36E-05 |
| DLG3 | -0.60103 | 7.50345 | 9.47E-05 |
| SCN3B | -0.96241 | 10.73489 | 9.50E-05 |
| CPNE4 | -1.06817 | 8.347833 | 9.55E-05 |
| PLD3 | -0.61673 | 9.67775 | 9.61E-05 |
| HIF3A | 0.643833 | 6.51011 | 0.000102 |
| THY1 | -0.61207 | 10.57108 | 0.000102 |
| VAC14-AS1 | 0.645331 | 5.615823 | 0.000103 |
| MFSD4A | -0.63147 | 7.831031 | 0.000104 |
| LINC01094 | 1.318246 | 7.04985 | 0.000104 |
| RFPL1S | -0.67114 | 8.837235 | 0.000105 |
| RPH3A | -0.97325 | 8.33368 | 0.00011 |
| SCG2 | -0.9326 | 8.835385 | 0.00011 |
| RIT2 | -0.92382 | 7.003372 | 0.000111 |
| NETO2 | -0.69941 | 6.996723 | 0.000111 |
| ABCA6 | 0.837443 | 4.793959 | 0.000114 |
| MS4A7 | 0.709436 | 5.945843 | 0.000118 |
| VSTM2A | -0.68373 | 7.122032 | 0.000118 |
| NMNAT2 | -0.62724 | 7.599988 | 0.000124 |
| CFAP126 | 0.86633 | 6.036194 | 0.000128 |
| MAP2K1 | -0.7399 | 10.90298 | 0.000129 |
| GEM | 0.977984 | 7.296906 | 0.000139 |
| DPY19L2P2 | -0.60238 | 7.434241 | 0.000143 |
| PAX6 | 0.60389 | 7.270662 | 0.000143 |
| SLC16A6 | -0.63625 | 6.181077 | 0.000164 |
| FGF12 | -0.90608 | 7.127152 | 0.000165 |
| FAXC | -0.65909 | 6.361044 | 0.000169 |
| SCG3 | -0.6064 | 7.913837 | 0.00017 |
| YAP1 | 0.678192 | 6.859802 | 0.00017 |
| KCNV1 | -0.75327 | 6.004243 | 0.000173 |
| ARHGEF9 | -0.58922 | 8.529427 | 0.000176 |
| MAP2K4 | -0.6677 | 8.964252 | 0.000183 |
| C2orf80 | -0.85388 | 8.326061 | 0.000184 |
| STX1A | -0.6506 | 8.910018 | 0.00019 |
| C3AR1 | 0.652105 | 7.670342 | 0.000191 |
| SOX9 | 0.589025 | 9.752101 | 0.000193 |
| ID3 | 0.829106 | 7.816239 | 0.000194 |
| NPTX1 | -0.80141 | 10.20053 | 0.000214 |
| APLNR | 0.922958 | 9.562678 | 0.000224 |
| SLC32A1 | -0.70205 | 6.652739 | 0.000226 |
| NUPR1 | 0.706992 | 8.055834 | 0.000232 |
| HSPB8 | 0.690254 | 8.482157 | 0.000238 |
| EMP3 | 0.63177 | 7.24133 | 0.00024 |
| SYN1 | -0.76154 | 9.551789 | 0.000244 |
| TMEM158 | -0.68201 | 9.126232 | 0.000255 |
| BDNF | -1.01896 | 6.86221 | 0.000257 |
| CHRM1 | -0.85781 | 8.144636 | 0.000258 |
| C1orf216 | -0.59579 | 9.879465 | 0.000268 |
| NECAB1 | -0.96196 | 7.227881 | 0.000268 |
| LOC100506563 | -0.63285 | 6.171646 | 0.000269 |
| CRYM | -0.93984 | 9.129135 | 0.000278 |
| UNC5C-AS1 | -0.71127 | 6.362934 | 0.000283 |
| CACNA2D3 | -0.67941 | 8.498615 | 0.000292 |
| PLK2 | -0.87742 | 9.329794 | 0.00032 |
| CPLX1 | -0.77179 | 9.937909 | 0.000325 |
| ARPC1A | -0.58649 | 9.523541 | 0.000354 |
| CXCR4 | 0.884919 | 6.660329 | 0.000361 |
| GRAMD2B | 0.598789 | 8.276786 | 0.000374 |
| CARTPT | -0.70735 | 8.238603 | 0.000388 |
| SEMA3E | -0.64156 | 5.495124 | 0.000395 |
| PPP3R1 | -0.65328 | 7.03518 | 0.000396 |
| CCK | -0.98514 | 9.491813 | 0.000396 |
| SCN2A | -0.63077 | 9.685569 | 0.000399 |
| PTH2R | -0.72059 | 5.733216 | 0.000404 |
| RNASE4 | 0.679352 | 5.594618 | 0.000412 |
| CDH12 | -0.88233 | 6.191941 | 0.000415 |
| ZNF385B | -0.75824 | 5.875085 | 0.000418 |
| LAMB1 | -0.68904 | 5.470507 | 0.000418 |
| PTPN20 | -0.61813 | 4.344547 | 0.000432 |
| VAT1L | -0.63831 | 8.385327 | 0.000451 |
| CD163 | 1.012713 | 6.089045 | 0.000453 |
| TMEM130 | -0.69117 | 9.286213 | 0.000454 |
| CD44 | 0.646531 | 5.820886 | 0.000468 |
| NELL2 | -0.81737 | 11.64301 | 0.000474 |
| GMPR | 0.77781 | 7.528907 | 0.000483 |
| TPD52L1 | 0.604357 | 9.04649 | 0.000497 |
| MYBPC1 | 0.629463 | 8.765418 | 0.0005 |
| LRRTM1 | -0.67988 | 7.787829 | 0.000504 |
| GALNT15 | 0.71876 | 8.474017 | 0.000513 |
| RTN4RL1 | -0.58623 | 7.542283 | 0.000526 |
| PHYHD1 | 0.728239 | 7.680865 | 0.000528 |
| CFI | 0.724098 | 5.951178 | 0.00054 |
| ERICH3 | -0.81275 | 6.968523 | 0.000548 |
| NRGN | -0.76937 | 11.13727 | 0.000548 |
| B3GALT2 | -0.61166 | 7.165525 | 0.000548 |
| MAFB | 0.625914 | 7.720425 | 0.000551 |
| SLC16A9 | 0.806913 | 8.479042 | 0.000569 |
| SLITRK3 | -0.60491 | 7.689807 | 0.000581 |
| SUSD4 | -0.80434 | 7.675542 | 0.000581 |
| AEBP1 | 0.846744 | 8.137714 | 0.000585 |
| SMIM17 | -0.73046 | 7.697978 | 0.000592 |
| SYNGR3 | -0.71742 | 9.749059 | 0.000594 |
| CRH | -0.59635 | 5.959925 | 0.000608 |
| ZDHHC23 | -0.7168 | 7.387667 | 0.000614 |
| CRLF1 | 0.648849 | 7.364527 | 0.000614 |
| LTF | 1.065758 | 6.498666 | 0.000659 |
| EVI2B | 0.636863 | 6.396972 | 0.000718 |
| ACTR3B | -0.62782 | 8.169339 | 0.000778 |
| PCSK2 | -0.63618 | 8.646711 | 0.000821 |
| TRIM22 | 0.637177 | 7.802199 | 0.000826 |
| ADRA2A | -0.61525 | 6.307328 | 0.00085 |
| AKR1C3 | 0.647406 | 7.518668 | 0.000889 |
| RASL12 | 0.766966 | 7.990088 | 0.000915 |
| NRN1 | -0.67334 | 10.69327 | 0.000929 |
| INSYN2B | -0.60725 | 5.787026 | 0.000973 |
| PTPN3 | -0.60139 | 6.722652 | 0.00099 |
| TSPAN6 | 0.625995 | 7.570924 | 0.000998 |
| ZCCHC12 | -0.86565 | 8.724573 | 0.001044 |
| HERC2P3 | 0.694214 | 6.128202 | 0.001044 |
| C1orf87 | 0.592722 | 4.991243 | 0.001047 |
| GALNT17 | -0.66134 | 9.331262 | 0.001082 |
| SERPINI1 | -0.60646 | 10.40792 | 0.001161 |
| GRP | -0.84054 | 6.087542 | 0.001177 |
| TMEM35A | -0.74197 | 8.77435 | 0.001188 |
| FGF13 | -0.81411 | 8.866998 | 0.001228 |
| UBE2QL1 | -0.75015 | 9.862922 | 0.001247 |
| UNC5D | -0.66569 | 6.962468 | 0.001255 |
| GRIN2A | -0.64502 | 8.203297 | 0.001296 |
| EMX2 | 0.624373 | 8.032974 | 0.001329 |
| C1R | 0.616332 | 7.300033 | 0.001353 |
| RCAN2 | -0.64477 | 9.588552 | 0.001372 |
| ARRDC4 | 0.639434 | 8.429859 | 0.001379 |
| S100A11 | 0.62284 | 6.707907 | 0.001387 |
| FSTL5 | -0.63249 | 7.381766 | 0.001405 |
| ARMC3 | 0.590018 | 5.076445 | 0.001432 |
| MYOT | 0.766458 | 7.000127 | 0.001487 |
| KCNA1 | -0.59479 | 7.793299 | 0.001599 |
| TMEM45A | -0.58989 | 5.541135 | 0.001612 |
| TRHDE | -1.11614 | 7.325953 | 0.001665 |
| WIF1 | -0.79488 | 7.725091 | 0.001669 |
| RAB3B | -0.74629 | 6.104427 | 0.001703 |
| SERPINA3 | 1.432045 | 9.781904 | 0.001758 |
| LAMP5 | -0.72377 | 8.996384 | 0.001769 |
| CRHBP | -0.64992 | 6.123742 | 0.001792 |
| EPCAM | -0.63789 | 6.255915 | 0.001822 |
| KCNJ6 | -0.59443 | 8.402559 | 0.001845 |
| TLR2 | 0.668386 | 6.812892 | 0.001864 |
| ZFPM2 | -0.67407 | 8.482775 | 0.001989 |
| NPY | -0.87343 | 8.40914 | 0.00217 |
| HSPB1 | 0.72063 | 9.710998 | 0.002181 |
| NRIP3 | -0.88268 | 9.891569 | 0.002317 |
| ENC1 | -0.70221 | 10.91098 | 0.002413 |
| LINC01354 | 0.640883 | 5.088164 | 0.002484 |
| TALAM1 | 0.587445 | 8.840773 | 0.002485 |
| SCIN | 0.649654 | 6.022085 | 0.002587 |
| RTN4RL2 | -0.68342 | 5.666024 | 0.002593 |
| SLC47A2 | 0.883489 | 5.915028 | 0.002606 |
| CNIH3 | -0.63411 | 7.144972 | 0.002627 |
| HLA-DRA | 0.938107 | 8.397538 | 0.002867 |
| SLC14A1 | 0.978664 | 7.567862 | 0.002876 |
| RSPO2 | -0.62726 | 4.857618 | 0.002972 |
| COL21A1 | 0.591718 | 6.771745 | 0.003087 |
| HTR2C | -0.91005 | 5.910924 | 0.003139 |
| PRKCG | -0.58546 | 7.547259 | 0.003285 |
| CBLN2 | -0.66138 | 7.670971 | 0.00329 |
| ZMAT4 | -0.60438 | 7.18208 | 0.003301 |
| GABRA1 | -0.86168 | 9.112544 | 0.003304 |
| GBP3 | 0.771869 | 5.91854 | 0.003388 |
| DACH2 | -0.59457 | 8.032643 | 0.003474 |
| GPR22 | -0.65771 | 6.01216 | 0.003532 |
| PDYN | -0.96588 | 7.050205 | 0.003625 |
| AQP4 | 0.693114 | 9.17823 | 0.003727 |
| VSIG4 | 0.865654 | 7.593265 | 0.00376 |
| TAFA1 | -0.76558 | 8.595807 | 0.003762 |
| PPM1E | -0.85325 | 8.60946 | 0.004063 |
| HCLS1 | 0.781173 | 7.629739 | 0.004619 |
| PIRT | 0.682972 | 7.243292 | 0.004856 |
| GABRA5 | -0.8367 | 6.804633 | 0.00508 |
| HLA-DPA1 | 0.697426 | 7.488497 | 0.005117 |
| PCSK1 | -0.97346 | 7.280145 | 0.005117 |
| NEFH | -0.70523 | 8.599508 | 0.005211 |
| SLC17A6 | -1.2491 | 6.547687 | 0.005434 |
| RGS4 | -1.03217 | 8.377926 | 0.005574 |
| TMEM125 | -0.58632 | 8.08658 | 0.005994 |
| NEUROD6 | -0.65568 | 8.852108 | 0.006396 |
| PCP4 | -0.79896 | 9.260999 | 0.006455 |
| TBR1 | -0.67194 | 5.960108 | 0.006534 |
| OPALIN | -0.61131 | 9.216938 | 0.006745 |
| PVALB | -0.95766 | 5.954226 | 0.007436 |
| SOSTDC1 | -0.64487 | 6.106145 | 0.007948 |
| PTPRT | -0.59288 | 7.423676 | 0.007981 |
| FCGBP | 0.630067 | 7.660232 | 0.007981 |
| BAG3 | 0.59492 | 9.452167 | 0.008541 |
| SERTM1 | -0.87348 | 6.77187 | 0.008663 |
| GRIA1 | -0.6901 | 8.537423 | 0.009203 |
| SPARC | 0.591387 | 10.51847 | 0.009367 |
| C1QC | 0.751618 | 8.135651 | 0.009428 |
| HLA-DMA | 0.598262 | 7.511045 | 0.009597 |
| LINC01088 | 0.905437 | 6.817796 | 0.009755 |
| PCDH8 | -0.95598 | 9.015014 | 0.011262 |
| C1QA | 0.668346 | 8.083052 | 0.011315 |
| CCN1 | 0.674357 | 7.257783 | 0.011605 |
| SLC17A7 | -0.66288 | 10.74218 | 0.01174 |
| EGR3 | -0.60451 | 8.423038 | 0.012716 |
| SLC39A12 | 0.628709 | 8.319599 | 0.014757 |
| RNASE6 | 0.595633 | 6.463473 | 0.014969 |
| FKBP5 | 0.689654 | 8.262618 | 0.01556 |
| SERPINF1 | -0.65546 | 8.082507 | 0.015664 |
| MAFF | 0.725715 | 7.295185 | 0.017172 |
| ZFP36 | 0.613961 | 8.982216 | 0.021606 |
| MT1M | 0.634734 | 9.340183 | 0.021828 |
| S100A12 | 0.667921 | 4.616062 | 0.021962 |
| FOS | 0.753561 | 8.224894 | 0.023679 |
| CHI3L1 | 0.648396 | 7.556304 | 0.024373 |
| RGS1 | 0.6906 | 5.397724 | 0.024788 |
| C3 | 0.586821 | 9.610418 | 0.025328 |
| AQP1 | 0.73553 | 9.010627 | 0.026857 |
| RXFP1 | -0.68804 | 5.060794 | 0.026915 |
| OR7A5 | 0.598251 | 6.698011 | 0.028683 |
| FAM81B | 0.664647 | 4.649522 | 0.030113 |
| C1QB | 0.682403 | 7.731588 | 0.031224 |
| AZGP1 | 0.610784 | 5.597084 | 0.047894 |

Table 2.107 AD-related DEGs

| Symbol | Log_2_FC | AveExpression | adj.P.Val |
| --- | --- | --- | --- |
| GABRG2 | -1.29834 | 8.5616 | 1.77E-05 |
| ATP1A3 | -1.27774 | 8.836432 | 1.32E-05 |
| NEFL | -1.07388 | 9.783472 | 3.07E-05 |
| RGS4 | -1.03217 | 8.377926 | 0.005574 |
| BDNF | -1.01896 | 6.86221 | 0.000257 |
| TUBA4A | -0.99256 | 10.11261 | 1.02E-06 |
| SYT1 | -0.97447 | 11.05236 | 2.65E-05 |
| PCSK1 | -0.97346 | 7.280145 | 0.005117 |
| PDYN | -0.96588 | 7.050205 | 0.003625 |
| SCN3B | -0.96241 | 10.73489 | 0.000095 |
| SYN2 | -0.94095 | 7.61468 | 8.3E-06 |
| CRYM | -0.93984 | 9.129135 | 0.000278 |
| MRAP2 | -0.93491 | 7.827956 | 5.62E-05 |
| GABRD | -0.91059 | 7.321332 | 2.72E-05 |
| KCNQ5 | -0.90961 | 6.115755 | 7.41E-05 |
| FGF12 | -0.90608 | 7.127152 | 0.000165 |
| NECAP1 | -0.88778 | 9.546775 | 2.16E-06 |
| MLLT11 | -0.88569 | 11.8331 | 1.02E-06 |
| MDH1 | -0.88424 | 11.8139 | 2.08E-06 |
| EEF1A2 | -0.86877 | 10.72831 | 7.5E-06 |
| ATP6V1B2 | -0.86639 | 10.92996 | 1.46E-05 |
| GABRA1 | -0.86168 | 9.112544 | 0.003304 |
| GABBR2 | -0.85482 | 9.388268 | 1.03E-05 |
| GLRB | -0.84464 | 7.995211 | 2.02E-05 |
| SNX10 | -0.84203 | 8.588832 | 3.09E-05 |
| ELOVL4 | -0.84095 | 7.77513 | 2.16E-06 |
| GABRA5 | -0.8367 | 6.804633 | 0.00508 |
| TUBB3 | -0.79728 | 11.19398 | 6.31E-07 |
| ATP6V1A | -0.77958 | 9.719419 | 5.19E-05 |
| RASGRP1 | -0.77887 | 8.917278 | 2.37E-05 |
| CPLX1 | -0.77179 | 9.937909 | 0.000325 |
| SCN2B | -0.76818 | 7.762376 | 6.55E-06 |
| REEP1 | -0.76418 | 9.589363 | 7.43E-06 |
| SYN1 | -0.76154 | 9.551789 | 0.000244 |
| ATP8A2 | -0.75785 | 8.252351 | 2.91E-06 |
| UBE2QL1 | -0.75015 | 9.862922 | 0.001247 |
| MAP2K1 | -0.7399 | 10.90298 | 0.000129 |
| SYP | -0.7292 | 8.726456 | 1.45E-05 |
| TUBB2A | -0.70748 | 12.49273 | 6.31E-07 |
| NEFH | -0.70523 | 8.599508 | 0.005211 |
| SLC7A14 | -0.69939 | 6.988239 | 2.66E-05 |
| SNAP25 | -0.69873 | 10.80515 | 2.23E-05 |
| BBS7 | -0.69337 | 6.932926 | 5.27E-06 |
| TRAPPC6B | -0.69308 | 7.847749 | 1.77E-05 |
| XK | -0.69012 | 6.86747 | 0.00007 |
| LAMB1 | -0.68904 | 5.470507 | 0.000418 |
| SLC12A5 | -0.68313 | 10.34278 | 2.95E-05 |
| ZFPM2 | -0.67407 | 8.482775 | 0.001989 |
| TBR1 | -0.67194 | 5.960108 | 0.006534 |
| GAD1 | -0.66867 | 6.886129 | 6.35E-05 |
| CDK5 | -0.66809 | 8.960037 | 1.35E-05 |
| UBE2T | -0.6675 | 6.254891 | 1.88E-05 |
| GPI | -0.6639 | 10.50407 | 2.41E-06 |
| GALNT17 | -0.66134 | 9.331262 | 0.001082 |
| SERPINF1 | -0.65546 | 8.082507 | 0.015664 |
| ACTL6B | -0.65135 | 7.566793 | 8.94E-05 |
| PTPRN | -0.64578 | 7.976715 | 9.01E-05 |
| GRIN2A | -0.64502 | 8.203297 | 0.001296 |
| NUP93 | -0.64254 | 7.496113 | 8.72E-07 |
| EPCAM | -0.63789 | 6.255915 | 0.001822 |
| SCN2A | -0.63077 | 9.685569 | 0.000399 |
| RSPO2 | -0.62726 | 4.857618 | 0.002972 |
| MAGEL2 | -0.62569 | 7.265276 | 0.000011 |
| OPCML | -0.62286 | 9.608385 | 4.11E-05 |
| TUSC3 | -0.62169 | 6.426824 | 9.36E-05 |
| ATP6V1E1 | -0.62018 | 10.9412 | 6.31E-07 |
| TUBB4B | -0.61774 | 11.17988 | 1.66E-06 |
| PLD3 | -0.61673 | 9.67775 | 9.61E-05 |
| ATP1A1 | -0.6135 | 10.64921 | 5.94E-05 |
| MDH2 | -0.61265 | 9.115255 | 2.41E-06 |
| SERPINI1 | -0.60646 | 10.40792 | 0.001161 |
| DLG3 | -0.60103 | 7.50345 | 9.47E-05 |
| ARL6 | -0.59908 | 6.179431 | 4.79E-05 |
| KCNA1 | -0.59479 | 7.793299 | 0.001599 |
| KCNJ6 | -0.59443 | 8.402559 | 0.001845 |
| MKKS | -0.58951 | 8.126865 | 1.02E-06 |
| ARHGEF9 | -0.58922 | 8.529427 | 0.000176 |
| SLC25A12 | -0.58867 | 8.555733 | 2.33E-05 |
| PRKCG | -0.58546 | 7.547259 | 0.003285 |
| C3 | 0.586821 | 9.610418 | 0.025328 |
| SOX9 | 0.589025 | 9.752101 | 0.000193 |
| SPARC | 0.591387 | 10.51847 | 0.009367 |
| BAG3 | 0.59492 | 9.452167 | 0.008541 |
| PAX6 | 0.60389 | 7.270662 | 0.000143 |
| EMX2 | 0.624373 | 8.032974 | 0.001329 |
| DTNA | 0.625006 | 8.838374 | 5.73E-05 |
| MAFB | 0.625914 | 7.720425 | 0.000551 |
| MYBPC1 | 0.629463 | 8.765418 | 0.0005 |
| BCL6 | 0.634982 | 7.929246 | 7.01E-05 |
| CHI3L1 | 0.648396 | 7.556304 | 0.024373 |
| CRLF1 | 0.648849 | 7.364527 | 0.000614 |
| TNFRSF11B | 0.65058 | 4.0091 | 6.05E-06 |
| TLR5 | 0.655057 | 6.81517 | 5.22E-06 |
| NOTCH2NLA | 0.661636 | 9.128583 | 0.000024 |
| C1QA | 0.668346 | 8.083052 | 0.011315 |
| YAP1 | 0.678192 | 6.859802 | 0.00017 |
| HSPB8 | 0.690254 | 8.482157 | 0.000238 |
| HSPB1 | 0.72063 | 9.710998 | 0.002181 |
| CFI | 0.724098 | 5.951178 | 0.00054 |
| VCAN | 0.724317 | 8.094979 | 1.11E-05 |
| C1QC | 0.751618 | 8.135651 | 0.009428 |
| MYOT | 0.766458 | 7.000127 | 0.001487 |
| GLIS3 | 0.787527 | 6.73598 | 4.75E-05 |
| AEBP1 | 0.846744 | 8.137714 | 0.000585 |
| PRKX | 0.863381 | 6.706232 | 2.76E-05 |
| CXCR4 | 0.884919 | 6.660329 | 0.000361 |
| CP | 1.318213 | 6.839914 | 5.29E-05 |

Table 3. Top10 AD-related DEGs by 3 topological analysis methods of CytoHubba.

| MCC | EPC | Degree |
| --- | --- | --- |
| SNAP25 | SNAP25 | SNAP25 |
| SLC12A5 | GAD1 | GAD1 |
| SYN1 | GABRA1 | GABRA1 |
| SYP | SLC12A5 | SYN1 |
| BDNF | SYN1 | BDNF |
| GRIN2A | GABRG2 | NEFL |
| SYT1 | SCN2A | GRIN2A |
| GAD1 | GRIN2A | GABRG2 |
| NEFL | SYT1 | SCN2A |
| GABRA1 | NEFL | SLC12A5 |

Table 4. Variation importance of the top20 genes obtained by randomForest algorithm

| Gene | Importance of variation |
| --- | --- |
| ATP6V1G2 | 1.965086 |
| ATP1A3 | 1.315839 |
| GABRG2 | 0.690101 |
| VSNL1 | 0.683305 |
| NWD2 | 0.550604 |
| SST | 0.549253 |
| SMIM43 | 0.524786 |
| AMPH | 0.452435 |
| CD163 | 0.382539 |
| MAL2 | 0.372462 |
| NAP1L5 | 0.327229 |
| SYNPR | 0.302692 |
| SERPINA3 | 0.291664 |
| BDNF | 0.271816 |
| STMN2 | 0.269239 |
| CALB1 | 0.245084 |
| LTF | 0.242423 |
| CHGB | 0.240995 |
| CP | 0.227716 |
| RGS4 | 0.21846 |

Table 5. ROC curve analysis of top10 hub genes

| Genes | AUC | CI |
| --- | --- | --- |
| SYN1 | 0.841 | 0.700-0.982 |
| SNAP25 | 0.784 | 0.569-0.999 |
| GABRA1 | 0.693 | 0.439-0.947 |
| SYT1 | 0.795 | 0.618-0.973 |
| GRIN2A | 0.773 | 0.592-0.953 |
| GABRG2 | 0.767 | 0.557-0.978 |
| SLC12A5 | 0.818 | 0.654-0.983 |
| SYP | 0.869 | 0.720-1.000 |
| KCNA1 | 0.739 | 0.518-0.959 |
| GABRD | 0.562 | 0.288-0.837 |

Table 6. ROC curve analysis of top20 hub genes recognized from random forest analysis

| Genes | AUC | CI |
| --- | --- | --- |
| ATP6V1G2 | 0.864 | 0.723-1.000 |
| GABRG2 | 0.767 | 0.557-0.978 |
| AMPH | 0.818 | 0.654-0.982 |
| VSNL1 | 0.824 | 0.657-0.991 |
| NWD2 | 0.625 | 0.376-0.874 |
| SST | 0.602 | 0.343-0.861 |
| SMIM43 | 0.455 | 0.200-0.709 |
| AMPH | 0.818 | 0.654-0.982 |
| CD163 | 0.511 | 0.256-0.766 |
| MAL2 | 0.722 | 0.491-0.953 |
| NAP1L5 | 0.705 | 0.503-0.906 |
| SYNPR | 0.648 | 0.439-0.857 |
| SERPINA3 | 0.767 | 0.570-0.964 |
| BDNF | 0.835 | 0.690-0.981 |
| STMN2 | 0.847 | 0.695-0.999 |
| CALB1 | 0.784 | 0.622-0.946 |
| LTF | 0.784 | 0.599-0.969 |
| CHGB | 0.864 | 0.725-1.000 |
| CP | 0.812 | 0.589-1.000 |
| RGS4 | 0.869 | 0.728-1.000 |

## Supplementary Figures


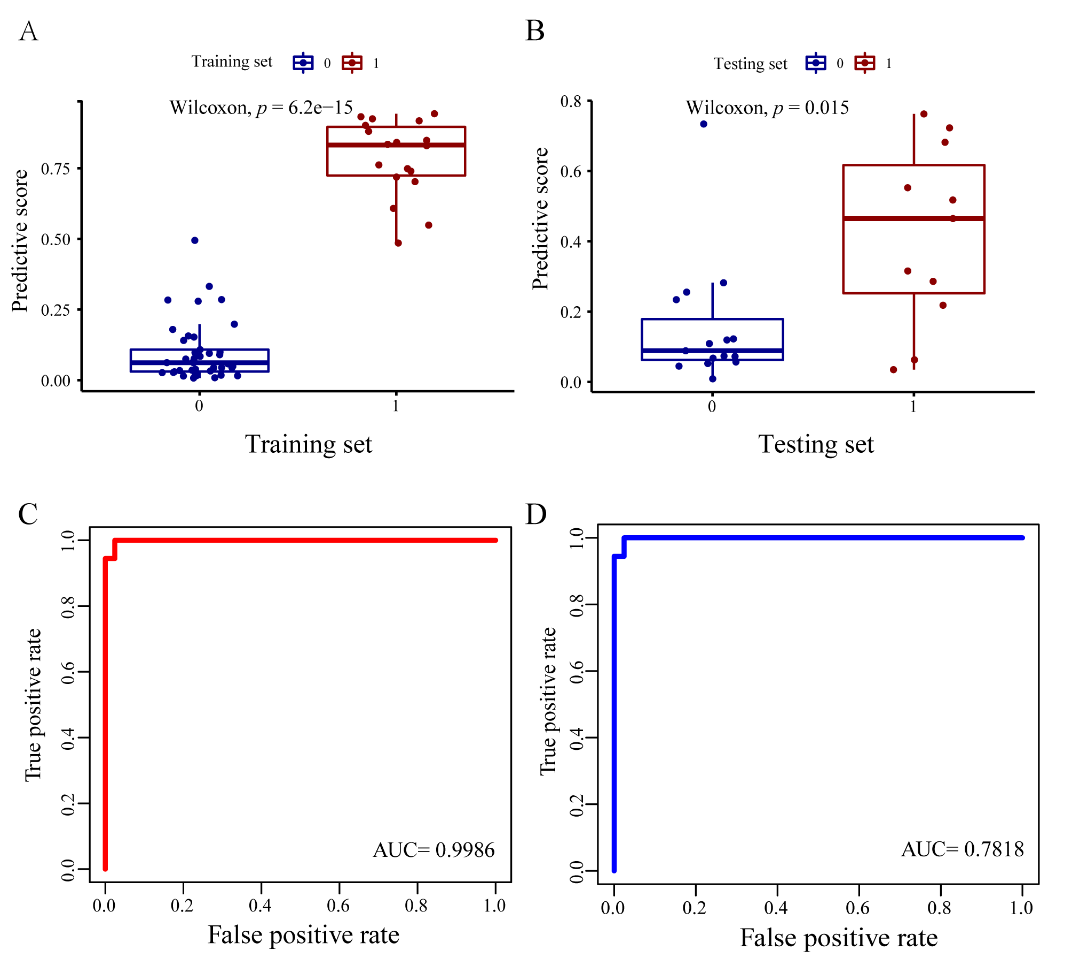


**Supplementary Figure 1.** Evaluating the accuracy of randomForest predictive model. (A, B) Predictive scores for diagnosis of training set and test set. (C, D) ROC curves for evaluating the accuracy of model in training set and test set.
